# Unsaturated Fatty Acids Are Decreased in Aβ Plaques in Alzheimer's Disease

**DOI:** 10.1111/jnc.16306

**Published:** 2025-01-18

**Authors:** Dominik Röhr, Melina Helfrich, Marcus Höring, Frederik Großerüschkamp, Gerhard Liebisch, Klaus Gerwert

**Affiliations:** ^1^ Center for Protein Diagnostics (PRODI) Biospectroscopy, Ruhr University Bochum Bochum Germany; ^2^ Department of Biophysics Faculty of Biology and Biotechnology, Ruhr University Bochum Bochum Germany; ^3^ Institute of Clinical Chemistry and Laboratory Medicine, University Hospital Regensburg Regensburg Germany

**Keywords:** Alzheimer's disease, amyloid‐beta plaques, fatty acids, infrared spectroscopy, laser microdissection, lipidomics, mass spectrometry, neural networks, neurodegeneration, oxidative stress

## Abstract

Alzheimer's disease (AD) is characterized by the accumulation of amyloid‐beta (Aβ) plaques in the brain, contributing to neurodegeneration. This study investigates lipid alterations within these plaques using a novel, label‐free, multimodal approach. Combining infrared (IR) imaging, machine learning, laser microdissection (LMD), and flow injection analysis mass spectrometry (FIA‐MS), we provide the first comprehensive lipidomic analysis of chemically unaltered Aβ plaques in post‐mortem human AD brain tissue. IR imaging revealed decreased lipid unsaturation within plaques, evidenced by a reduction in the alkene (=C‐H) stretching vibration band. The high spatial resolution of IR imaging, coupled with machine learning‐based plaque detection, enabled precise and label‐free extraction of plaques via LMD. Subsequent FIA‐MS analysis confirmed a significant increase in short‐chain saturated lipids and a concomitant decrease in long‐chain unsaturated lipids within plaques compared to the surrounding tissue. These findings highlight a substantial depletion of unsaturated fatty acids (UFAs) in Aβ plaques, suggesting a pivotal role for lipid dysregulation and oxidative stress in AD pathology. This study advances our understanding of the molecular landscape of Aβ plaques and underscores the potential of lipid‐based therapeutic strategies in AD.

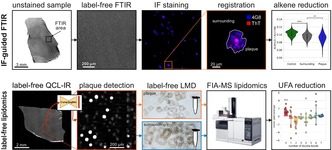

## Graphical abstract

Alzheimer's disease is characterized by amyloid‐beta (Aβ) plaque accumulation and neurodegeneration. This study reveals decreased levels of unsaturated fatty acids (UFAs) in plaques from post‐mortem AD brain tissue. Using Fourier‐transform infrared (FTIR) spectroscopy, we detected a reduction in alkene bands specific to UFAs. With precise localization by quantum cascade laser infrared (QCL‐IR) microscopy and laser microdissection (LMD), lipidomic analysis via liquid chromatography‐mass spectrometry (LC‐MS) confirmed elevated short‐chain saturated and depleted long‐chain unsaturated lipids in plaques. These findings underscore lipid dysregulation within Aβ plaques, suggesting UFAs as potential therapeutic targets in AD.

**Abbreviations**
AD: Alzheimer's disease
Aβ: amyloid‐beta
Cer: ceramides
CNN: convolutional neural network
EMSC: extended multiplicative signal correction
FA: fatty acid
FIA‐FTMS: flow injection analysis Fourier transform mass spectrometry
FIA‐MS: flow injection analysis mass spectrometry
FIA‐MS/MS: flow injection analysis‐tandem mass spectrometry
FOV: field of view
FTIR: Fourier‐transform infrared
HC: healthy control
HexCer: hexosylceramides
HNE: 4‐hydroxy‐2‐nonenal
IF: Immunofluorescence
IHC: immunohistochemistry
IR: infrared
LC‐MS: liquid chromatography mass spectrometry
LMD: laser microdissection
LPC: lysophosphatidylcholines
LPE: lysophosphatidylethanolamine
MALDI‐IMS: matrix‐assisted laser desorption/ionization imaging mass spectrometry
MALDI‐TOF: matrix‐assisted laser desorption ionization‐time of flight
MDA: malondialdehyde
MTL: middle temporal lobe
NA: numerical aperture
NBB: Netherlands Brain Bank
NIA‐AA: National Institute on Aging‐Alzheimer's Association
PBS: phosphate‐buffered saline
PC: phosphatidylcholine
PCA: principal component analysis
PE: phosphatidylethanolamine
PFA: paraformaldehyde
PI: phosphatidylinositol
PS: phosphatidylserine
PUFA: polyunsaturated fatty acid
QCL‐IR: quantum cascade laser infrared
ROS: reactive oxygen species
SFA: saturated fatty acid
SM: sphingomyelins
SNR: signal‐to‐noise ratio
STL: superior temporal lobe
SVD: singular value decomposition
SVM: support vector machine
ThT: Thioflavin T
TL: temporal lobe
UFA: unsaturated fatty acid
WSI: whole‐slide image

## 1 Introduction

Alzheimer's disease (AD) is a neurodegenerative disorder, hallmarked by the deposition of Aβ peptides in the brain. Aβ aggregates into β‐sheet‐rich oligomers and fibrils (Kirschner et al. 1987; Michaels et al. 2020). These aggregated forms of Aβ continually accumulate in the intercellular space and drive the development of Aβ plaques in the brain (Rozemuller et al. 1989; Ikeda et al. 1990; Thal et al. 2006; Röhr et al. 2020). Aβ's interactions with surrounding tissues are complex and actively researched. Aβ, in its various aggregated forms, engages in dynamic interactions with adjacent cells, extracellular matrix components, and other proteins, shaping the microenvironment and influencing the progressive pathogenesis of AD (Hardy and Higgins 1992).

Aβ's interaction with the lipid membranes of adjacent cells has been indicated as a potential mediator of toxicity (Arispe, Pollard, and Rojas 1994; Markesbery 1997). The amphipathic nature of Aβ allows it to integrate into lipid bilayers, disrupting their structural integrity. This perturbation, in turn, influences ion channel conductance and cellular homeostasis, contributing to synaptic dysfunction and neuronal damage (Arispe, Rojas, and Pollard 1993; Lin, Bhatia, and Lal 2001; Fantini et al. 2014; Ciudad et al. 2020). Furthermore, Aβ, especially in its aggregated form, has the capacity to directly induce oxidative stress in lipid membranes (Behl et al. 1994; Butterfield and Sultana 2011; Butterfield, Swomley, and Sultana 2013). The proposed mechanism involves the interaction of Aβ with metal ions and the generation of reactive oxygen species (ROS) (Smith, Cappai, and Barnham 2007; Butterfield et al. 2010). Additionally, Aβ may induce oxidative stress indirectly, by inducing mitochondrial dysfunction or by interfering with the antioxidant defense system (Reddy and Beal 2008; Butterfield, Swomley, and Sultana 2013).

The abundance of highly oxidizable unsaturated fatty acids (UFAs) in the brain makes it particularly vulnerable to oxidative stress (Prasad et al. 1998). Oxidants abstract weak allylic hydrogens from UFAs, creating alkyl radicals. By addition of molecular oxygen, a peroxyl radical is formed, which can, in turn, abstract another allylic hydrogen from a nearby UFA. Thus, an FA hydroperoxide and another alkyl radical are produced. The latter can start the reaction cycle anew (Girotti 1985). This process, called lipid peroxidation, can propagate through lipid membranes, producing instable FA hydroperoxides. The FA hydroperoxides are cleaved, resulting in shorter, less unsaturated FAs and byproducts, which are detrimental for neuronal functions (Reiter 1995; Markesbery and Lovell 1998; Di Paolo and Kim 2011).

The composition of FAs is known to be altered in the AD brain compared to the healthy control (HC) brain (Corrigan et al. 1998; Prasad et al. 1998; Fraser, Tayler, and Love 2010). This is particularly relevant because FAs play a central role in maintaining the fluidity and structural integrity of cell membranes (Di Paolo and Kim 2011). Notably, Nakada et al. revealed significantly decreased levels of UFAs in phospholipids in the temporal lobe (TL) of AD brains, compared to HC brains (Nakada, Kwee, and Ellis 1990). Likewise, Söderberg et al. reported decreased levels of UFAs in the major phospholipids phosphatidylethanolamine (PE) and phosphatidylcholine (PC) in multiple AD brain regions (Söderberg et al. 1991). In both studies, the reduction in UFAs was paralleled by increased levels of shorter saturated FAs (SFAs).

The evidence of decreased UFAs in AD tissue has led to the hypothesis that Aβ‐mediated oxidative stress causes UFA degradation in Aβ plaques (Markesbery 1997). Garcia‐Alloza et al. observed increased levels of ROS in Aβ plaques of a transgenic AD mouse model using in vivo multi‐photon microscopy (Garcia‐Alloza et al. 2006). Likewise, Xie et al. reported markers of oxidative stress in neurons close to plaques and observed the resulting death of those neurons (Xie et al. 2013). This aligns with reports that neuronal damage follows plaque development in AD (Sheng et al. 1998; Xie et al. 2013; Kiskis et al. 2015). The cleavage byproducts of UFA hydroperoxides, such as 4‐hydroxy‐2‐nonenal (HNE), have been found to co‐localize with Aβ plaques (Ellis et al. 2010; Furman et al. 2016).

However, recent advances have significantly expanded our understanding of the lipid composition, spatially resolved in Aβ plaques. Several studies have investigated the lipid composition in plaques (Blank and Hopf 2020). Notably, Kaya et al. (2018) reported the accumulation of saturated PC and concurrent depletion of unsaturated PC in plaques a transgenic AD mouse model. Similarly, Michno et al. employed multimodal imaging techniques to reveal both the decrease in unsaturated phosphoserines (PS) and PE, along with Aβ aggregation‐dependent lipid alterations in plaques, demonstrating the distinct accumulation of ceramides in cored plaques and phosphatidylinositols (PI) in diffuse plaques (Michno et al. 2018, 2022). While these studies provide valuable insights, investigations into the lipidome of Aβ plaques in human AD cases have been limited. Notably, Panchal et al. employed mass spectrometry to analyze laser microdissected plaques and surrounding tissue, reporting elevated levels of saturated ceramides (Cer) and cholesterol within plaques (Panchal et al. 2010, 2014). Most recently, mass spectrometry imaging has been instrumental in elucidating the lipid composition of plaques (Michno et al. 2024). Coupled with machine learning, it uncovered significant heterogeneity in lipid profiles between amyloid‐positive individuals with and without cognitive impairment (Enzlein et al. 2024). Furthermore, Huang et al. revealed the accumulation of lysophospholipids and ceramides (Cer) around Aβ plaques in advanced Braak stage AD brain tissue. This suggests spatially heterogeneous lipid changes, similar to those observed in transgenic mouse models (Huang et al. 2024).

A significant limitation for the use of LMD extracted plaques is the necessity to perform immunohistochemical (IHC) staining prior to LMD. IHC requires the incubation of tissue sections in solvents as well as the application of reagents for fixation and blocking (Westermark, Johnson, and Westermark 1999). Depending on the exact IHC protocol, lipids may be dislodged from the samples during the staining procedure. Most recently, our group demonstrated the significant loss of soluble proteins in plaques during anti‐Aβ IHC (Müller et al. 2024).

In this study, we used Fourier transform infrared (FTIR) imaging to investigate the degree of lipid unsaturation in Aβ plaques. This label‐free technique spatially resolves the distribution of chemical groups, such as the alkenes in UFAs, prior to the invasive IHC staining. We further apply label‐free quantum cascade laser infrared (QCL‐IR) imaging to detect and extract Aβ plaques in fresh frozen AD brain tissue sections. This novel method generates highly precise and chemically native plaque samples in sufficient quantities for mass spectrometric analysis (Müller et al. 2024). This allowed us to fully harbor the main advantage of mass spectrometry to detect and quantify a wide range of lipid species with high accuracy and reproducibility (Schwudke et al. 2007; Blank and Hopf 2020). The result is a comprehensive insight into the native plaque lipidome that allowed us to uncover a systematic depletion of long UFAs in plaques.

## 2 Methods

### 2.1 Case Selection

Human brain tissue for this non‐pre‐registered study was obtained post‐mortem from the Netherlands Brain Bank (NBB), Netherlands Institute for Neuroscience, Amsterdam in accordance with strict ethical guidelines. The Institutional Review Board and Medical Ethical Board from Vrije University Medical Center Amsterdam approved the procedures of the NBB. All materials have been collected from donors for or from whom a written informed consent for brain autopsy and the use of the material and clinical information for research purposes had been obtained by NBB. The neuropathological diagnosis was performed using standardized procedures, with AD cases (*n* = 8) meeting the National Institute on Aging‐Alzheimer's Association (NIA‐AA) criteria for AD (Montine et al. 2012). No formal sample size calculations were performed. The number of samples was granted from the NBB upon request. HC cases (*n* = 8) were chosen based on the lack or low presence of AD pathology, indicated by their ABC scores, see Table 1, and no reported cognitive decline during life. To reduce confounding variables, the AD and HC groups were matched by age and sex. A detailed overview of individual patient information can be found in Table S1.

**TABLE 1:** Details on group demographics and neuropathological staging. Neuropathological scoring for Aβ deposits (A), neurofibrillary tangles (B), and neuritic plaques (C) (Montine et al. 2012).

	*n*	Sex (M/F)	Age	A 0/1/2/3	B 0/1/2/3	C 0/1/2/3
AD	8	5/3	88 ± 10	0/0/1/7	0/1/2/5	0/0/4/4
HC	8	4/4	89 ± 9	3/5/0/0	0/3/5/0	8/0/0/0

Abbreviations: AD, Alzheimer's disease; F, female; HC, healthy control; M, male.

### 2.2 Brain Tissue Preparation

Tissue samples from AD and HC were exclusively obtained from the TL, as this region is associated with Aβ plaque pathology in AD. Specifically, the samples were taken from the superior TL (STL) and middle TL (MTL), with details of the individual regions provided in Table S1. Tissue sections (10 μm) were thaw‐mounted on 1.4 μm Leica PET frame slides for vibrational imaging and subsequent anti‐Aβ IF or LMD and FIA‐MS. To preserve Aβ plaques and lipid changes in AD, samples were stored at −80°C until experimentation.

### 2.3 FTIR Imaging

FTIR microspectroscopy was performed in transmission mode with a Cary 670 spectrometer coupled to a Cary 620 microscope (Agilent Technologies) according to established protocols (Röhr et al. 2020). The microscope features a nitrogen‐cooled focal plane array (FPA) detector with 128 × 128 elements and a 15× (0.62 NA) objective. The instrument can achieve a nominal pixel size of 1.1 μm at 5× optical magnification. The 128 × 128‐element data acquisition yields a field of view (FOV) of around 141 × 141 μm^2^. Interferograms were acquired with 128 scans, resulting in a spectral range of 3700–948 cm^−1^ at a spectral sampling interval of 1.9 cm^−1^, using Blackman‐Harris‐4‐term apodization, power phase correction, and zero‐filling factor 2 for Fourier transform. Background correction was carried out by measuring the clean area of each CaF_2_ slide (512 scans) and subtracting it from the sample measurements. The software Resolutions Pro 5.3 (Agilent Technologies) was used to facilitate image acquisition. Throughout the measurements, the instruments and sample cavity were continuously purged with dry air to reduce atmospheric water vapor contribution and keep the samples in a conserving dry state.

### 2.4 FTIR Spectral Data Analysis

A lipid consists of a head group and FAs. The FAs are methylene (CH_2_) chains ending in a methyl group (CH_3_). UFAs contain alkene groups with double‐bonded carbon atoms (H‐C=C‐H). The =C‐H stretching vibration of these alkenes produces a distinct vibrational band 3012 cm^−1^ in infrared (IR) absorbance spectra (Socrates 2001; Dreissig et al. 2009). The intensity of the alkene band can be used to assess the degree of unsaturation of FAs (Yoshida and Yoshida 2003). The FAs are bound to the head groups by ester groups that feature a characteristic C=O stretching vibration band at 1738 cm^−1^ (Socrates 2001). The ester band serves as proxy for total lipid content (Robinson et al. 2022). Here, the absorbance ratio A_3012_ /A_1738_ is used to calculate the degree of lipid unsaturation. All calculations were performed in Python 3.10, utilizing NumPy 1.26.3, Pandas 1.5.3, and scikit‐learn 1.4.2. Significance levels were assessed using Welch's t‐test without adjustments for multiple comparisons, as only two independent groups were compared. For visualization, Statannotations 0.6 (Charlier 2022) was used. Confidence levels were determined based on *p*‐values: < 0.05 (*), < 0.01 (**), < 0.001 (***), and < 0.0001 (****). Before analysis, FTIR spectra were thoroughly prepared to ensure data quality and reliability, as described previously (Röhr et al. 2020). In brief, to avoid statistical distortions caused by outlier spectra, spectra with high noise level (signal‐to‐noise‐ration SNR < 100) and high scattering were excluded from analysis. The remaining spectra were then corrected for Mie scattering using extended multiplicative signal correction (EMSC). This correction method effectively reduces the effects of Mie scattering, which can complicate spectral interpretation (Konevskikh, Lukacs, and Kohler 2018; Solheim et al. 2019). An average spectrum of a plaque core has been created by manually selecting and averaging fitting pixel‐spectra. This was done for illustration spectral differences. All plots were created with matplotlib 3.8.4 and seaborn 0.11.2.

### 2.5 Immunofluorescence (IF) Staining

**FIGURE 1:**
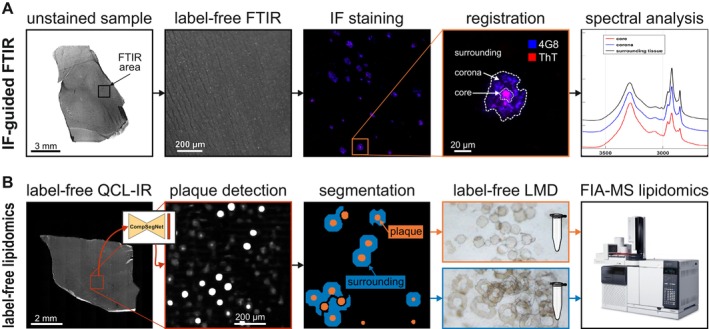
Workflows for our label‐free approaches. (A) Workflow for immunofluorescence (IF) guided Fourier transform infrared (FTIR) imaging. Unstained brain tissue sections are first imaged with FTIR subsequently stained against Aβ and scanned. Both modalities are registered, and region‐specific FTIR spectra are analyzed. (B) Unstained brain tissue sections are first imaged with QCL‐IR, and plaques are detected using a neural network (Müller et al. 2024). Plaques and surrounding tissue areas are extracted via laser microdissection (LMD) and analyzed using flow injection analysis mass spectrometry (FIA‐MS).

Anti‐Aβ IHC was performed using a multi‐label direct IF approach, staining samples with two anti‐Aβ probes with varying affinities for different Aβ polymorphs; see Figure 1A. The goal was to distinguish Aβ fibrils from oligomeric forms without using any reagents that could alter the structure of Aβ. The samples were fixed in 4% paraformaldehyde (PFA) for 30 min and then washed with phosphate‐buffered saline (PBS). Samples were incubated with 20 μM Thioflavin T (ThT, Thermo Fisher) in 2% mouse serum (Novus Biologicals) in PBS for 45 min, washed in PBS, and then incubated overnight at 4°C with 4G8, labeled with Spark‐YG 570 (BioLegend) (1:1000) in normal antibody diluent (DAKO). Following additional washing, samples were mounted with EverBrite TrueBlack Hardset mounting medium (Biotium) and cover‐slipped. The stained sample was then imaged with an Olympus VS120 slide scanner and the UPlanSApo 20× 0.75 NA (numerical aperture) objective (Olympus). The rigorous protocol visualized Aβ using fluorescent labels ThT and 4G8.

### 2.6 Multimodal Image Registration

To link spectral data with IF images, custom software (written in Matlab 2019a) was used to determine an affine 2D transformation, as described previously (Röhr et al. 2020). The user‐provided reference coordinates were used to transform the IF image into the vibrational image's coordinate system. The overlay's quality was visually confirmed while accounting for tissue morphology. To identify spectra corresponding to Aβ plaques, binary masks were generated from IF image cutouts using Otsu's method, delineating plaques, and surrounding regions. The resulting plaque spectrum was calculated as the arithmetic mean of all pixel‐spectra within the plaque mask, and the surrounding spectrum was derived from the pixel‐spectra within a ring‐shaped mask that surrounded the plaque; see Figure 1A.

### 2.7 QCL‐IR Imaging

QCL‐IR was used to generate microscopic spectral images of the tissue sections intended for the label‐free detection and extraction of plaques via LMD for FIA‐MS. The Spero‐QT 340 microspectrometer (Daylight Solutions, San Diego, USA) and Chemical Vision software version 3.2 were used for this purpose. This label‐free method employs infrared spectra, which contain useful chemical information in the form of vibrational bands (Goormaghtigh et al. 2010; Röhr et al. 2020). The spectral range of the instrument is 1800 to 948 cm^−1^, with a spectral resolution of 2 cm^−1^. A 4× objective (0.3 NA) was used to project a 2 × 2 mm^2^ FOV onto a 480 × 480 pixel microbolometer FPA detector, yielding an image with 4.25 × 4.25 μm^2^ pixel size. Prior to spectral measurements, tissue samples were thawed in a dry air‐filled container before being placed in the Spero‐QT cavity. Continuous purging with dry air was used throughout the measurements.

### 2.8 Aβ Plaque Detection Using a Neural Network

In our previous work (Müller et al. 2024), we detailed the label‐free detection of plaques in QCL‐IR images using a deep convolutional neural network (CNN). In summary, we used a comparative segmenting network (CompSegNet) trained for Aβ plaque detection (Schuhmacher, Schörner, and Küpper 2022). The network's architecture is based on an extended U‐Net model with input dimensions of 64 × 64 × 427 pixels (Ronneberger, Fischer, and Brox 2015). QCL‐IR images are cropped to 64 × 64 pixels and then passed through convolution and pooling layers, which gradually reduce the image dimensions to an 8 × 8 × 512 matrix. Transposed convolutions, combined with skip‐connections, are then applied, resulting in the generation of a 64 × 64 × 1 output layer called the activation map. To facilitate whole‐slide image (WSI) segmentation, a tile‐based strategy was used, with a fixed window size of 64 × 64 pixels. Overlapping tiles of 16 pixels were created to ensure information continuity across adjacent regions, and the CNN evaluated each tile individually. Finally, the outputs from each tile were reassembled using the maximum value for overlapping regions, yielding a comprehensive WSI activation map that identifies the plaques; see Figure 1B.

### 2.9 Plaque Extraction via Laser Microdissection (LMD)

LMD is used to extract plaques from brain tissue sections, guided by the WSI activation maps, as described previously (Müller et al. 2024). In brief, by binarizing the activation maps with a predetermined threshold of 0.9 within a range of 0 to 1, “plaque masks” were created. Morphological operations were performed sequentially, excluding objects smaller than 100 μm^2^, dilation by 15 μm, hole filling, erosion by 10 μm, and exclusion of objects smaller than 300 μm^2^, based on eccentricity and solidity criteria; see Figure 1B. The morphological processing and coordinate transformation were done in Matlab (version 2019a), resulting in plaque‐like shapes with a 5 μm margin to account for tissue loss during subsequent procedures. This results in plaques that are approximately spherical in shape, with areas exceeding 300 μm^2^. Following manual exclusion of damaged tissue regions, the sample was laser microdissected with a LMD microscope (PALM MicroBeam; Zeiss, Jena, Germany). A two‐dimensional Helmert transformation, which used reference points in each microscopy image, facilitated the plaque shape coordinates to the microscopy coordinate system. The PALM Robo software (Zeiss, version 4.6) was then used to import the coordinates and perform precise plaque extraction with the instrument's 20× objective. Extracted tissue samples were collected in 50 mM SDS, briefly sonified, covered with argon gas, and stored at −80°C until FIA‐MS analysis. In general, several thousand shapes were combined to reach a total area of 50 million μm^2^ per sample. This procedure was applied to plaques, their surrounding regions, as well as control tissue.

### 2.10 Sample Preparation for FIA‐MS

Lipid extraction was performed according to the procedure described by Bligh and Dyer (Bligh and Dyer 1959). A sample amount of 50 μg wet weight was subjected to lipid extraction in the presence of non‐naturally occurring internal standards. The following lipid species were added as internal standards: CE 17:0, CE 22:0, FC[D7], Cer 18:1;O2/14:0, Cer 18:1;O2[D7]/18:0, HexCer 18:1;O2/12:0, HexCer 18:1;O2[D5]/18:0, DG 14:0/14:0, DG 20:0/20:0, FC[D7], LPC 13:0, LPC 19:0, LPE 13:0, LPE 18:1[D7], PC 14:0/14:0, PC 22:0/22:0, PE 14:0/14:0, PE 20:0/20:0 (di‐phytanoyl), PI 18:1[D7]/15:0, PS 14:0/14:0, PS 20:0/20:0 (di‐phytanoyl), SM 18:1;O2/12:0, SM 18:1;O2/18:1[D9], TG 17:0/17:0/17:0, and TG 19:0/19:0/19:0. After extraction, a volume of 800 μL of the chloroform phase (total volume of 1 mL) was recovered by a pipetting robot and vacuum dried. The dried extracts were dissolved in 500 μL chloroform/methanol/2‐propanol (1:2:4 v/v/v) with 7.5 mM ammonium formate.

### 2.11 FIA‐MS Lipidomics

The analysis of lipids was performed by direct FIA using a triple quadrupole mass spectrometer (FIA‐MS/MS) and a high‐resolution hybrid quadrupole‐Orbitrap mass spectrometer (FIA‐FTMS). FIA‐MS/MS was performed in positive ion mode using the analytical setup and strategy described previously (Liebisch et al. 2004). A Waters Acquity UPLC (Milford, Massachusetts, USA) delivered methanol/chloroform = 3/1 (v/v) with 7.5 mM ammonium acetate at an initial flow rate of 50 μL/min for 0.2 min, followed by 10 μL/min for 5.7 min and a wash at 500 μL/min for 1.9 min. The LC flow was coupled to a Waters Xevo TQ‐S micro equipped with an electrospray ionization source operated in positive mode. A fragment ion of m/z 184 was used for lysophosphatidylcholines (LPC) (Liebisch et al. 2002). The following neutral losses were applied: PE and lysophosphatidylethanolamine (LPE) 141, phosphatidylserine (PS) 185, and phosphatidylinositol (PI) 277 (Matyash et al. 2008). Sphingosine‐based Cer and hexosylceramides (HexCer) were analyzed using a fragment ion of m/z 264 (Liebisch et al. 1999). For FIA‐MS/MS glycerophospholipid species, annotation is based on the assumption of even numbered carbon chains only. A detailed description of the FIA‐FTMS method was published recently (Höring et al. 2020, 2021). QExactive Orbitrap (Thermo Fisher Scientific, Bremen, Germany) equipped with a heated electrospray ionization source was used with the following settings: spray voltage of 3.5 kV, S‐lens RF level 50, capillary temperature of 250°C, aux gas heater temperature of 100°C, and settings of 15 for sheath gas and 5 for aux gas. Chloroform/methanol/2‐propanol (1:2:4 v/v/v) was delivered at an initial flow rate of 100 μL/min until 0.25 min followed by 10 μL/min for 2.5 min and a wash out with 300 μL/min for 0.5 min. PC, PC ether (PC O) and sphingomyelins (SM) were analyzed in negative ion mode m/z 520–960 as [M+HCOO]‐ at a target resolution of 140 000 (at m/z 200). The quantification was performed by multiplication of the spiked IS amount with analyte‐to‐IS ratio. Lipid species were annotated according to the latest proposal for shorthand notation of lipid structures that are derived from mass spectrometry (Liebisch et al. 2020).

### 2.12 Lipidomics Data Analysis

Lipidomic analysis by mass spectrometry yields molar concentrations of lipid species for each sample. Here, each sample was normalized for the total molar lipid content to compare their lipid composition. Lysophospholipids were excluded from analysis because they contain only one FA and can therefore not be compared with the other lipid species investigated here.

For the principal component analysis (PCA), standardized data with a mean of 0 and a standard deviation of 1 were utilized to ensure equal weighting of all features. The PCA was performed based on the correlation matrix, employing Singular Value Decomposition (SVD). All calculations were done in python 3.10, using numpy 1.26.3, pandas 1.5.3, and scikit‐learn 1.4.2. Significance levels were calculated using Welch's t‐test and visualized using Statannotations 0.6 (Charlier 2022). The confidence levels were determined according to *p*‐values < 0.05 (*), < 0.01 (**), < 0.001 (***), and < 0.0001 (****). All plots were created with matplotlib 3.8.4 and seaborn 0.11.2.

## 3 Results

### 3.1 Lipids Are Less Unsaturated in Plaques

Post‐mortem brain tissue sections from the TL of eight AD cases and eight HC cases were analyzed in this study. FTIR was performed on the selected gray matter regions within the tissue sections, followed by IF staining against Aβ.

**FIGURE 2:**
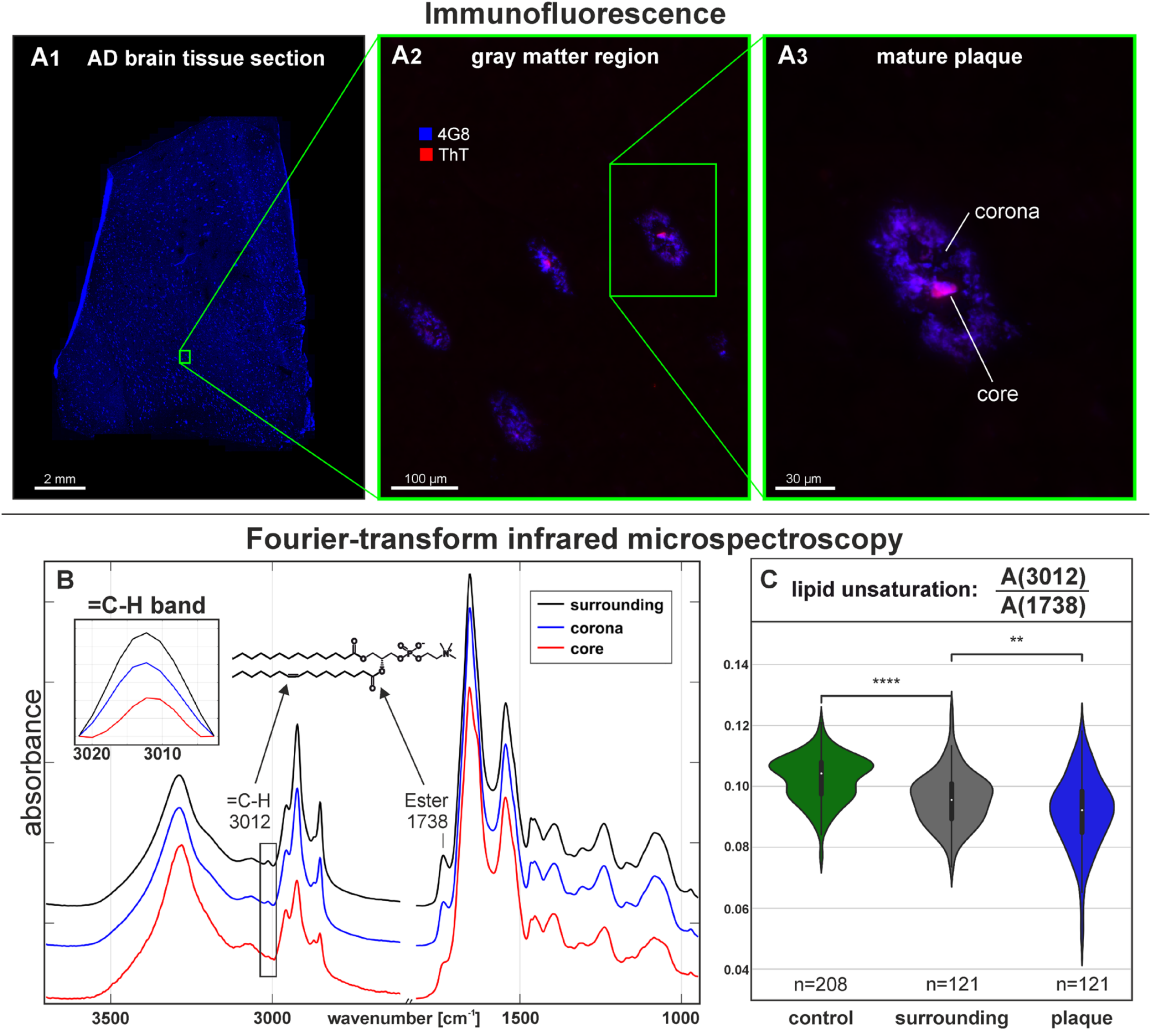
Lipid unsaturation in plaques in human brain tissue derived by immunofluorescence (IF) and Fourier‐transform infrared (FTIR) microspectroscopy. (A1) Tissue sections are stained against Aβ using the fluorophore‐labeled antibody 4G8 (blue) and the fluorescent probe Thioflavin T (ThT) in red. (A2) The gray matter region of an advanced AD TL (TL) is typically abundant in Aβ plaques. (A3) A mature plaque is readily identified in IF images by their characteristic core and corona structure. (B) The combination of FTIR and IF images of the same sample produce clearly separated mean FTIR spectra of a plaque core (red), corona (blue), and its surrounding tissue (black). The inset displays the marked decrease of the =C‐H stretching band of alkenes at 3012 cm^−1^ in the plaque. (C) The ratio between alkenes and ester groups (1738 cm^−1^) indicates the degree of lipid unsaturation and was calculated for 208 gray matter regions from three control cases and 121 plaques from three AD cases. Unsaturated lipids are significantly decreased in plaques, compared to their surrounding and gray matter of HC cases. The full statistical reports, including *p*‐values, *t*‐values, and degrees of freedom, are provided in Supporting Information S2. ** < 0.01, **** < 0.0001.

Figure 2 illustrates the decrease in lipid unsaturation within Aβ plaques, based upon the ratio between alkenes and ester groups. Figure 2A displays an exemplary brain tissue section from the TL of an AD case. IF against Aβ visualizes the distribution and microscopic details of plaques in the tissue section. By combining FTIR with subsequent IF, plaques were localized in FTIR images with micrometer precision, as described previously (Röhr et al. 2020). This facilitated the extraction and subsequent analysis of FTIR spectra from plaque core, corona, and its surrounding, as shown in Figure 2B. The reduced =C‐H stretching band of alkenes at 3012 cm^−1^ indicates a decrease of alkenes in plaques. Figure 2C displays that the ratio of the alkene band and the ester band at 1738 cm^−1^ is significantly decreased in plaques, compared to their surrounding tissue and gray matter of HC cases. This indicates lower levels of lipid unsaturation in plaques.

To further elucidate the specific lipidomic alterations associated with Aβ plaques, FIA‐MS was employed for in‐depth lipid profiling. Plaques were identified and localized in unstained tissue sections using fast, label‐free QCL‐IR imaging, circumventing potential artifacts introduced by staining methods. This non‐invasive approach, coupled with machine learning‐based plaque detection, enabled the precise microdissection of plaques and their surrounding tissue from tissue sections via LMD (Müller et al. 2024). This was done with brain tissue from all eight AD cases and gray matter from all HC cases.

**FIGURE 3:**
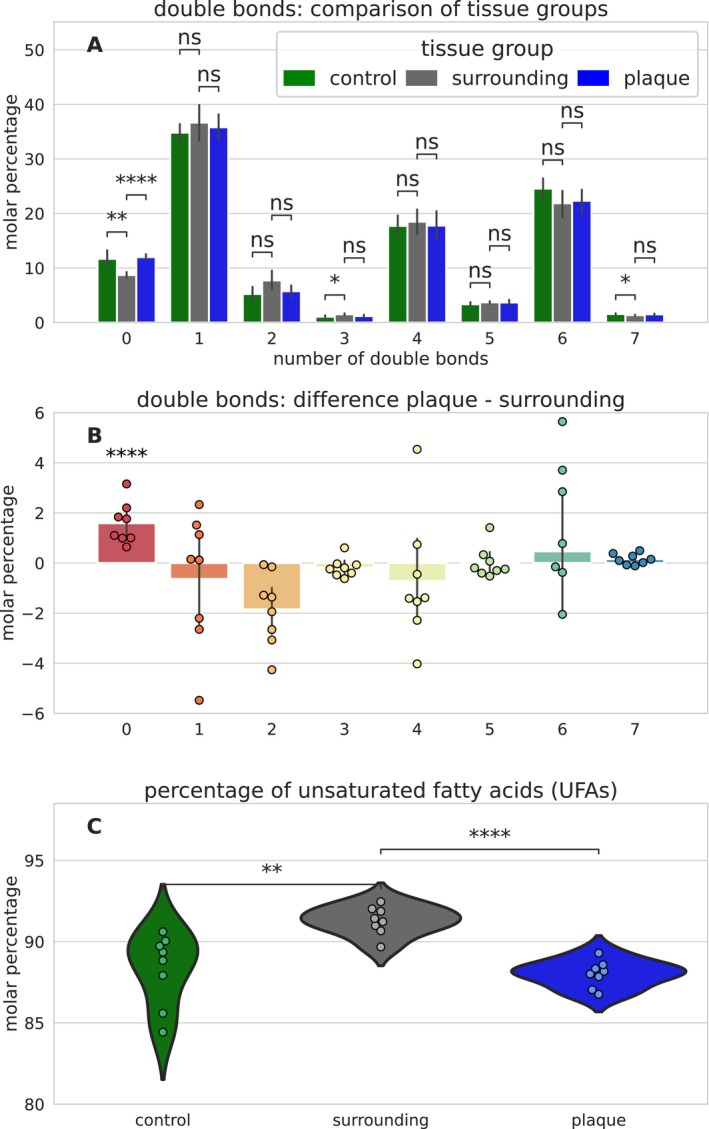
Lipid unsaturation derived by flow injection analysis mass spectrometry (FIA‐MS) of label‐free extracted Aβ plaques, surrounding tissue, and gray matter from healthy control (HC) cases. (A) The distribution of lipid unsaturation (number of C=C double bonds per lipid) across all samples (*n* = 24) from all cases (*n* = 16) and tissue groups as percentage of the total lipid content. Figure S4B illustrates the distribution of data on a patient‐wise basis. In some instances, the degree of lipid unsaturation differs significantly between tissue groups. (B) Subsequently, the relative content of unsaturated lipids differs significantly between the tissue groups and is lowest in plaques. (C) The distribution difference of lipid unsaturation between plaques and their surrounding tissue reveals that unsaturated lipids are significantly increased in plaques, whereas unsaturated lipids are decreased. The complete statistical reports, including *p*‐values, *t*‐values, and degrees of freedom, can be found in Supporting Information S2. ns > 0.05, ** < 0.01, **** < 0.0001.

Figure 3 shows the detailed lipid unsaturation profiles from FIA‐MS analysis. Figure 3A illustrates the composition of lipid unsaturation in all FIA‐MS samples (*n* = 24). Saturated lipids that contain only SFAs with no double bonds make up 11% ± 2% of the total lipid content. The residual lipids contain between one and seven double bonds. The most abundant species are monosaturated lipids that make up 36% ± 4%, whereas lipids with three double bonds are least abundant and make up 1.3% ± 0.4% of the lipid composition; see Figure S3A. The differences in lipid unsaturation in plaques, their surroundings, and gray matter from HC cases are most significant in saturated lipids. Consequently, the percentage of UFAs also differ significantly between the tissue groups, as shown in Figure 3B. HC tissue displays a broad range of UFA contents across the HC cases (*n* = 8), whereas plaques and their surrounding tissue from the AD cases (*n* = 8) feature tighter distributions. Presumably, this is the case because the extraction of plaques and their surroundings was guided by QCL‐IR imaging, whereas the HC tissue was collected from across the entire gray matter area. Plaques display the lowest UFA content (88% ± 1%), significantly lower than their surroundings (91% ± 1%). Figure 3C further illustrates the differences between plaques and their surroundings. Saturated lipids are significantly increased in plaques by 1.6% ± 0.5%. Unsaturated lipids with between one and four double bonds are decreased in plaques. Lipids with two double bonds display the strongest decrease by −1.8% ± 0.9%. Lipids with more than five double bonds remain broadly unchanged in plaques.

### 3.2 Fatty Acids Are Shorter in Plaques

**FIGURE 4:**
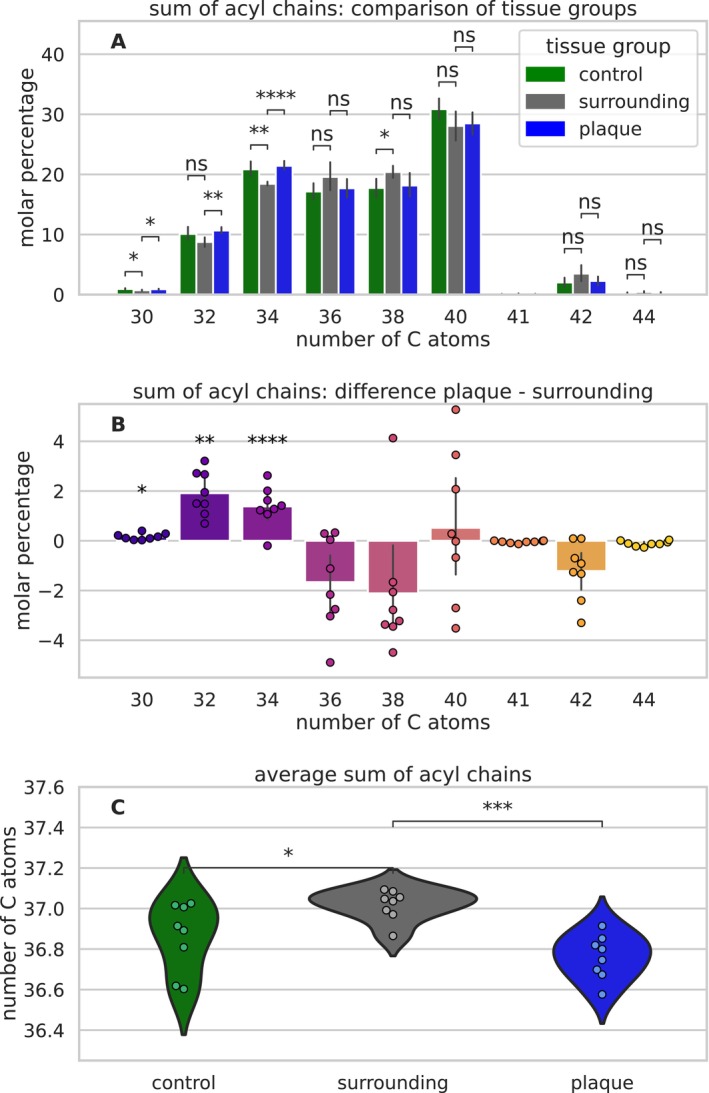
Sum of acyl chain composition derived by label‐free flow injection analysis mass spectrometry (FIA‐MS) of label‐free extracted Aβ plaques, surrounding tissue, and gray matter from healthy control (HC) cases. (A) The distribution of FA length (number of C atoms in FAs per lipid molecule) across all samples (*n* = 24) from all cases (*n* = 16) and tissue groups as percentage of the total lipid content. Figure S3D illustrates the distribution of data on a patient‐wise basis. The FA length distribution differs significantly between the tissue groups. (B) Subsequently, the average FA length differs significantly between the tissue groups and is lowest in plaques. (C) The distribution difference between plaques and their surrounding tissue reveals that short FAs (< 35 C atoms per lipid) are significantly increased in plaques, whereas long FAs (> 35 C atoms per lipid) are mostly decreased. The full statistical reports, including *p*‐values, *t*‐values, and degrees of freedom, are provided in Supporting Information S2. ns > 0.05, * < 0.05, ** < 0.01, *** < 0.001, **** < 0.0001.

The proposed cleavage of UFAs, described above, results in a length reduction of FAs. Figure 4 presents the FA length distribution differences between plaques, their surroundings, and HC tissue. Figure 4A displays the sum of acyl chain distribution in all our FIA‐MS samples (*n* = 24) given as the sum number of C atoms contained in both FAs in a lipid. The shortest detected FA pair yields a sum of 30 C atoms and is most likely a pair of a myristic acid (14:0) and a palmitic (16:0) or palmitoleic acid (16:1) and constitutes 0.85% ± 0.17% of the lipid composition; see Figure S3B. The longest FA pair we detected contains 44 C atoms and is most likely a combination of two C22 (22:x) FAs, because longer FAs are usually not detected in gray matter (Nakada, Kwee, and Ellis 1990; Söderberg et al. 1991; Fraser, Tayler, and Love 2010). The FA pairs with between 32 and 42 C atoms can contain various FA combinations and are correspondingly more abundant. The FA length distributions in plaques, their surroundings, and HC tissue display significant differences in the short FA pairs between 30 and 34 C atoms. Correspondingly, the average total FA length differs between the tissue groups, as shown in Figure 4B. HC tissue shows a broader range of FA length, compared to plaques and their surrounding tissue. This is similar to the lipid unsaturation shown above and presumably also due to the tissue collection procedure. The average FA length in plaques (36.76 ± 0.11 C) is significantly lower than in their surroundings (37.02 ± 0.07 C). Figure 4C details the differences in FA lengths between plaques and their surroundings. Short FA pairs (30–34 C) are significantly increased in plaques. FA pairs with 32 C atoms see the strongest increase by 1.9% ± 0.6%. All long FA pairs (36–44 C) are decreased in plaques, except C40 with a deviation much larger than the difference of 0.5% ± 1.8%.

### 3.3 PC Dominates the Lipidome Changes in Plaques

**FIGURE 5:**
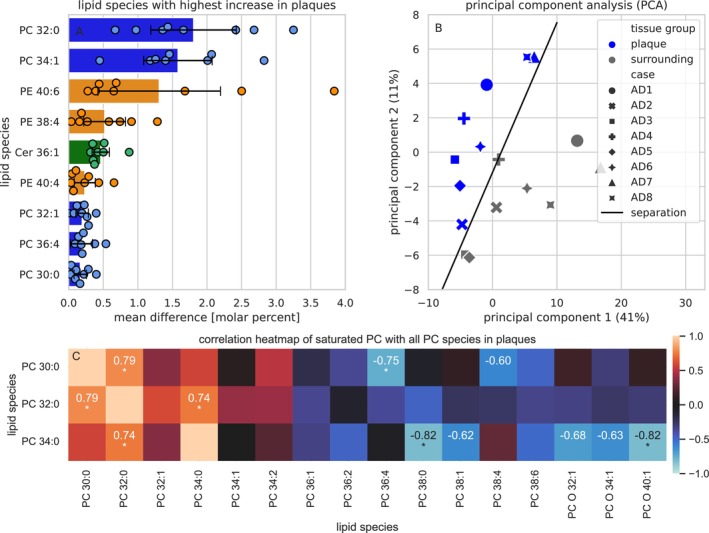
The lipid changes in plaques. (A) The mean difference of the total molar contributions of the most increased lipid species in plaques (*n* = 8), compared to their surroundings. Phosphatidylcholine (PC) in blue, PE in orange and Ceramides (Cer) in green. (B) Principal component analysis (PCA) of the entire lipidomes, comparing plaques (blue) and their surrounding (gray) from the eight AD cases included in this study. The black line indicates a linear separation of the cluster. (C) Correlation matrix between saturated and all PC species. Red indicates correlation, whereas blue indicates anti‐correlation. Strong correlations (*R* < 0.6) are labeled, and significant correlations (*p* > 0.05) are marked with an asterisk, excluding self‐correlations.

An analysis of the lipid species composition reveals that PC contributes strongest to the lipidome changes in plaques. Figure 5A displays the top nine contributors, five of which are PC species, three are PE species, and one is a Cer species. The strongest contributor is PC 32:0 that contributes 1.8% ± 0.6% more to the lipidome of plaques than to their surroundings. Figures S5 and S6 analyze PC separately and show that the reduction in lipid unsaturation and FA length is prominent in PC. A PCA reveals that the plaque lipidome differs systematically from the surrounding tissue in all AD cases (*n* = 8). This can be seen by the respective cluster (blue = plaque, gray = surrounding) in Figure 5B. Figure S7 displays the respective component loadings. The cluster are linearly separated by a separation line, determined by a support vector machine (SVM). Figure 5C presents a correlation matrix of the short and saturated PC species 30:0, 32:0, and 34:0 with all PC species in plaques. It is apparent that the short and saturated PC species broadly correlate with other short and (mono)saturated PC species (red hues, left side), whereas they anti‐correlate with long and unsaturated PC species (blue hues, right side). Correlations of all lipid species are shown in Figure S8.

## 4 Discussion

By employing FTIR, we were able to spatially resolve and quantify the unsaturation levels of lipids in Aβ plaques. Our findings indicate a significant reduction in the =C‐H stretching band at 3012 cm^−1^ of alkenes, a marker of unsaturated FAs, within plaques compared to surrounding tissue and HC brain regions. This approach provided a direct measurement of lipid unsaturation, circumventing the limitations associated with traditional oxidative stress markers. Many studies that have previously investigated oxidative stress relied on specific molecular markers to trace oxidative stress. However, common markers like 4‐hydroxylnonenal (HNE) and malondialdehyde (MDA) have been criticized for their lack of specificity, sensitivity, and reproducibility (Sultana, Perluigi, and Butterfield 2006). Further molecular details of the lipidomic alterations in plaques were elucidated using FIA‐MS, which confirmed that the fraction of unsaturated lipids is decreased in plaques. Specifically, we observed a decrease in long‐chain, unsaturated lipid species, compensated by a significant increase in short‐chain, saturated lipids in plaques.

Comparing our results to previous studies, the overall depletion of PUFAs in AD brains has been documented previously (Söderberg et al. 1991; Corrigan et al. 1998; Prasad et al. 1998; Fraser, Tayler, and Love 2010). Martín et al. reported reduced levels of PUFAs and a 13% decrease in alkenes in lipid rafts extracted from the frontal brain cortex of AD cases (Martín et al. 2010). Our observation of decreased alkenes within plaques suggests that lipid degradation in plaques significantly contributes to the overall decrease of alkenes in AD brains. However, our study extends this understanding by providing evidence that lipid degradation is specifically localized within Aβ plaques. This localized depletion highlights the direct impact of Aβ aggregation on lipid composition, differentiating our work from studies assessing global brain lipid levels without spatial specificity.

The spatially resolved lipid composition of plaques has only been investigated by a few studies so far. Panchal et al. reported increased levels of saturated Cer and cholesterol in plaques using FIA‐MS to compare plaques and surrounding tissue (Panchal et al. 2010, 2014). Our FIA‐MS analysis complements these findings by demonstrating a significant decrease in long‐chain UFAs within plaques. This suggests that while certain lipid species accumulate, others are selectively depleted, likely due to oxidative processes. Kaya et al. applied matrix‐assisted laser desorption/ionization imaging mass spectrometry (MALDI‐IMS) to Aβ plaques in transgenic mice, which offers high‐resolution insights into the distribution of specific lipid species within complex tissues, providing a comprehensive understanding of the lipid environment surrounding plaques. Therefore, depletion of UFA‐rich phospholipids was observed (Kaya et al. 2018). This observation corroborates our findings, as we also noted a significant reduction in UFA‐rich phospholipids within plaques. CARS imaging provides exceptionally high spatial resolution, enabling Kiskis et al. (2015) to investigate the microstructure of lipid changes in plaques. However, it is constrained by the limited information content of Raman bands. Our study specifically identified PC as a major contributor to this decrease, with a shift toward short‐chain, saturated PC species. Benseny‐Cases et al. used FTIR and reported the decrease of the 3012 cm^−1^ band in plaques of transgenic mice, indicating reduced lipid unsaturation (Benseny‐Cases et al. 2014). Surowka et al. similarly observed increased lipid oxidation in amyloid deposits in transgenic mouse brains (Surowka et al. 2017). This is supported by observations from Head et al., who found oxidized Aβ in 98% of plaque cores, indicating that oxidative stress is a central feature of plaque pathology (Head et al. 2001). These findings align well with our FTIR results on human brain samples, which also indicated a marked decrease in lipid unsaturation within Aβ plaques.

Despite significant findings, our study has several limitations. First, FTIR and FIA‐MS for lipid analysis have inherent constraints: FTIR spectral data can be affected by overlapping signals, and FIA‐MS, while sensitive, requires meticulous sample preparation and can be influenced by ion suppression and matrix effects. Additionally, our focus was on a limited number of lipid species, excluding glycolipids and minor lipids that could offer deeper insights into lipid alterations in AD (Blank and Hopf 2020). We measured total lipid mass, which leaves room for interpretation. However, our observed percentage of the shortest FA pair (30:x, made up of 14:0 and 16:x) at 0.85% ± 0.17% aligns well with literature values of approximately 0.8% ± 0.2% in human TL, validating our findings (Fraser, Tayler, and Love 2010). The observed lipid depletion, though statistically significant, had a small effect size, indicating subtle changes requiring highly sensitive detection methods. The spatial resolution of QCL‐IR imaging also poses a limitation. Although LMD allowed isolation and analysis of Aβ plaques, heterogeneity within plaques and their microenvironment could influence lipid composition and oxidative stress levels, potentially accounting for discrepancies with other studies (Gaudin et al. 2012). Plaque, surrounding, and control regions for FTIR analyses were selected based on IHC, while the areas chosen for LMD were unstained, given the label‐free nature of this approach. Control gray matter regions were manually selected based on QCL‐IR measurements, potentially accounting for the broader distribution observed within this subcohort. In contrast, plaque and surrounding areas in the QCL‐IR data were selected using machine learning. As demonstrated in our most recent publication, this label‐free detection of plaques via machine learning shows a high degree of concordance with established IHC methods (Müller et al. 2024). Nevertheless, certain discrepancies persist between the two techniques, which may have contributed to the variations discussed above. Additionally, using post‐mortem brain tissue introduces variability due to factors such as post‐mortem interval, preservation methods, and disease progression, potentially affecting in vivo accuracy. Our cross‐sectional study did not address temporal lipid changes throughout disease stages. Furthermore, other mechanisms such as mitochondrial dysfunction and neuroinflammation may also contribute to oxidative stress in the AD brain, adding further complexity to the observed lipid alterations. We did not investigate the functional causes and consequences of lipid depletion within Aβ plaques. While we observed a reduction in UFAs, its origin and its impact on neuronal function, membrane integrity, and synaptic activity remains unclear. Functional studies using cell culture or animal models could link our findings to specific pathological mechanisms and identify therapeutic targets.

To summarize, the relationship between Aβ pathology and overall lipid degradation has been well‐documented (Söderberg et al. 1991; Reiter 1995; Martín et al. 2010). Our study adds to this body of evidence by demonstrating that lipid degradation is not only a generalized phenomenon in AD brains but also a localized event within Aβ plaques. Our study has provided significant insights into the lipidomic landscape of Aβ plaques in AD, specifically highlighting the depletion of UFAs. By employing advanced techniques such as IR imaging and mass spectrometry, we were able to directly investigate the lipid composition within plaques, bypassing the limitations associated with traditional oxidative stress markers.

In conclusion, our study highlights the significance of lipid degradation within Aβ plaques, providing a more detailed understanding of the molecular changes occurring in AD. The localized depletion of UFAs within plaques underscores the critical role of lipid alterations in the pathogenesis of AD. Further research should include longitudinal studies and in vivo imaging techniques. This will be crucial for understanding the dynamic changes in lipid composition throughout AD progression and exploring potential therapeutic avenues targeting lipid metabolism.

## Author Contributions

**Dominik Röhr:** conceptualization, methodology, investigation, formal analysis, software, visualization, writing – original draft, writing – review and editing. **Melina Helfrich:** investigation, formal analysis, visualization, writing – review and editing. **Marcus Höring:** investigation, writing – review and editing, methodology. **Frederik Großerüschkamp:** project administration, methodology, writing – review and editing. **Gerhard Liebisch:** project administration, methodology, writing – review and editing. **Klaus Gerwert:** supervision, funding acquisition, writing – review and editing.

## Ethics Statement

The Institutional Review Board and Medical Ethical Board from the Vrije University Medical Center Amsterdam approved the procedures of the NBB. This project is registered at the NBB under project number 1430 and carries the title: “From plaque to CSF – and back again? An (un)expected journey of Amyloid‐β”.

## Consent

The brain samples were obtained from the Netherlands Brain Bank (NBB), Netherlands Institute for Neuroscience, Amsterdam. All materials have been collected from donors for or from whom a written informed consent for brain autopsy and the use of the material and clinical information for research purposes had been obtained by NBB.

## Conflicts of Interest

The authors declare no conflicts of interest.

## Supporting information


Data S1.

## Data Availability

The data that support the findings of this study are available from the corresponding author Klaus Gerwert upon reasonable request.
