# Supplementary material for: Unsaturated Fatty Acids Are Decreased in Aβ Plaques in Alzheimer's Disease
Source: J Neurochem. 2025 Jan 18;169(1):e16306. doi: 10.1111/jnc.16306 (PMC11742699; doi:10.1111/jnc.16306)
Supplement: Supplementary file 1 — Data S1. [file JNC-169-0-s001.docx]

Supplementary Material

**Unsaturated fatty acids are decreased in Aβ plaques in Alzheimer’s disease**

Dominik Röhr^1,2,+^ , Melina Helfrich^1,2,+^, Marcus Höring^3^, Frederik Großerüschkamp^1,2^, Gerhard Liebisch^3^, Klaus Gerwert^1,2^

^1^ Ruhr University Bochum, Center for Protein Diagnostics (PRODI), Biospectroscopy, Germany

^2^ Ruhr University Bochum, Faculty of Biology and Biotechnology, Department of Biophysics, Germany

^3^ Institute of Clinical Chemistry and Laboratory Medicine, University Hospital Regensburg, Germany

^+^ Shared first authors.

**Patient-specific information from the cohort**

| **Case** | **Age at Death (years)** | **Gender** | **Postmortem Delay (hours)** | **A** | **B** | **C** | **Brain Region** |
| --- | --- | --- | --- | --- | --- | --- | --- |
| **HC** |  |  |  |  |  |  |  |
| 1 | 76 | f | 07:15 | 0 | 1 | 0 | STL |
| 2 | 84 | f | 07:50 | 1 | 2 | 0 | STL |
| 3 | 102 | f | 03:55 | 1 | 2 | 0 | STL |
| 4 | 102 | f | 04:55 | 1 | 1 | 0 | STL |
| 5 | 79 | m | 06:20 | 1 | 1 | 0 | STL |
| 6 | 86 | m | 06:45 | 0 | 2 | 0 | STL |
| 7 | 87 | m | 06:20 | 0 | 2 | 0 | STL |
| 8 | 94 | m | 04:25 | 1 | 2 | 0 | STL |
| **AD** |  |  |  |  |  |  |  |
| 1 | 76 | f | 03:45 | 3 | 3 | 3 | MTL |
| 2 | 88 | f | 10:00 | 3 | 2 | 2 | STL |
| 3 | 98 | f | 06:05 | 3 | 3 | 2 | STL |
| 4 | 72 | m | 04:30 | 3 | 3 | 3 | STL |
| 5 | 77 | m | 09:10 | 2 | 1 | 2 | STL |
| 6 | 94 | m | 04:15 | 3 | 2 | 2 | STL |
| 7 | 97 | m | 05:10 | 3 | 3 | 3 | MTL |
| 8 | 98 | m | 04:45 | 3 | 3 | 3 | STL |

**Table S1. A detailed overview of individual patient data**, including age, sex, diagnosis, ABC score, clinical progression and analyzed brain region. Patients were selected based on the presence or absence of AD pathology and matched by age and sex with the control group. Neuropathological scoring for Aβ deposits (A), neurofibrillary tangles (B), and neuritic plaques (C) [Montine et al, 2012]. Abbreviations: AD: Alzheimer’s disease, HC: healthy control, m: male, f: female, MTL: middle temporal lobe, STL: superior temporal lobe.

**Full statistical reports**

| **Compared Groups** | **p-value** | **t-value** | **Degrees of freedom** |
| --- | --- | --- | --- |
| **Figure 2C: lipid unsaturation in plaques** | | |  |
| surrounding vs. plaque | .002 | 3.149 | 218.27 |
| control vs. surrounding | <.001 | 7.183 | 226.55 |
| **Figure 3A: double bonds: comparison of tissue groups** | | |  |
| 0: surrounding vs. plaque | <.001 | -7.782 | 29.85 |
| 0: control vs. surrounding | .006 | 3.542 | 19.58 |
| 1: surrounding vs. plaque | .710 | 0.381 | 26.48 |
| 1: control vs. surrounding | .399 | -0.879 | 22.83 |
| 2: surrounding vs. plaque | .111 | 1.72 | 25.82 |
| 2: control vs. surrounding | .057 | -2.090 | 27.35 |
| 3: surrounding vs. plaque | .071 | 1.952 | 29.69 |
| 3: control vs. surrounding | .030 | -2.43 | 28.01 |
| 4: surrounding vs. plaque | .712 | 0.377 | 29.45 |
| 4: control vs. surrounding | .639 | -0.479 | 28.62 |
| 5: surrounding vs. plaque | .923 | 0.100 | 20.50 |
| 5: control vs. surrounding | .171 | -1.462 | 24.15 |
| 6: surrounding vs. plaque | .799 | -0.260 | 29.60 |
| 6: control vs. surrounding | .136 | 1.588 | 28.28 |
| 7: surrounding vs. plaque | .104 | -1.753 | 27.23 |
| 7: control vs. surrounding | .026 | 2.519 | 27.74 |
| **Figure 3B: double bonds: difference plaque - surrounding** | | |  |
| 0 | <.001 | -7.782 | 29.85 |
| **Figure 3C: percentage of unsaturated fatty acids (UFAs)** | | |  |
| surrounding vs. plaque | <.001 | 7.782 | 29.85 |
| control vs. surrounding | .006 | -3.542 | 19.58 |
| **Figure 4A: sum of acyl chains: comparison of tissue groups** | | |  |
| 30: surrounding vs. plaque | .017 | -2.712 | 28.67 |
| 30: control vs. surrounding | .025 | 2.535 | 27.55 |
| 32: surrounding vs. plaque | .003 | -3.746 | 25.92 |
| 32: control vs. surrounding | .089 | 1.840 | 27.29 |
| 34: surrounding vs. plaque | <.001 | -7.156 | 20.95 |
| 34: control vs. surrounding | .006 | 3.706 | 17.28 |
| 36: surrounding vs. plaque | .246 | 1.218 | 26.37 |
| 36: control vs. surrounding | .128 | -1.644 | 23.78 |
| 38: surrounding vs. plaque | .116 | 1.722 | 21.66 |
| 38: control vs. surrounding | .027 | -2.522 | 25.54 |
| 40: surrounding vs. plaque | .801 | -0.257 | 28.87 |
| 40: control vs. surrounding | .098 | 1.788 | 26.71 |
| 42: surrounding vs. plaque | .167 | 1.481 | 23.68 |
| 42: control vs. surrounding | .102 | -1.781 | 24.14 |
| 44: surrounding vs. plaque | .289 | 1.108 | 26.65 |
| 44: control vs. surrounding | .177 | -1.443 | 23.71 |
| **Figure 4B: sum of acyl chains: difference plaque - surrounding** | | |  |
| 30 | .017 | -2.712 | 28.67 |
| 32 | .003 | -3.746 | 25.92 |
| 34 | <.001 | -7.156 | 20.95 |
| **Figure 4C: average sum of acyl chains** | | |  |
| surrounding vs. plaque | <.001 | 5.538 | 12.41 |
| control vs. surrounding | .039 | -2.389 | 9.58 |

**Table S2.** **Full statistical reports:** All analyses utilized the Welch t-test for the computation of p-values, t-values and degrees of freedom across all datasets.

**Overall composition of lipid unsaturation and sum of acyl chains**


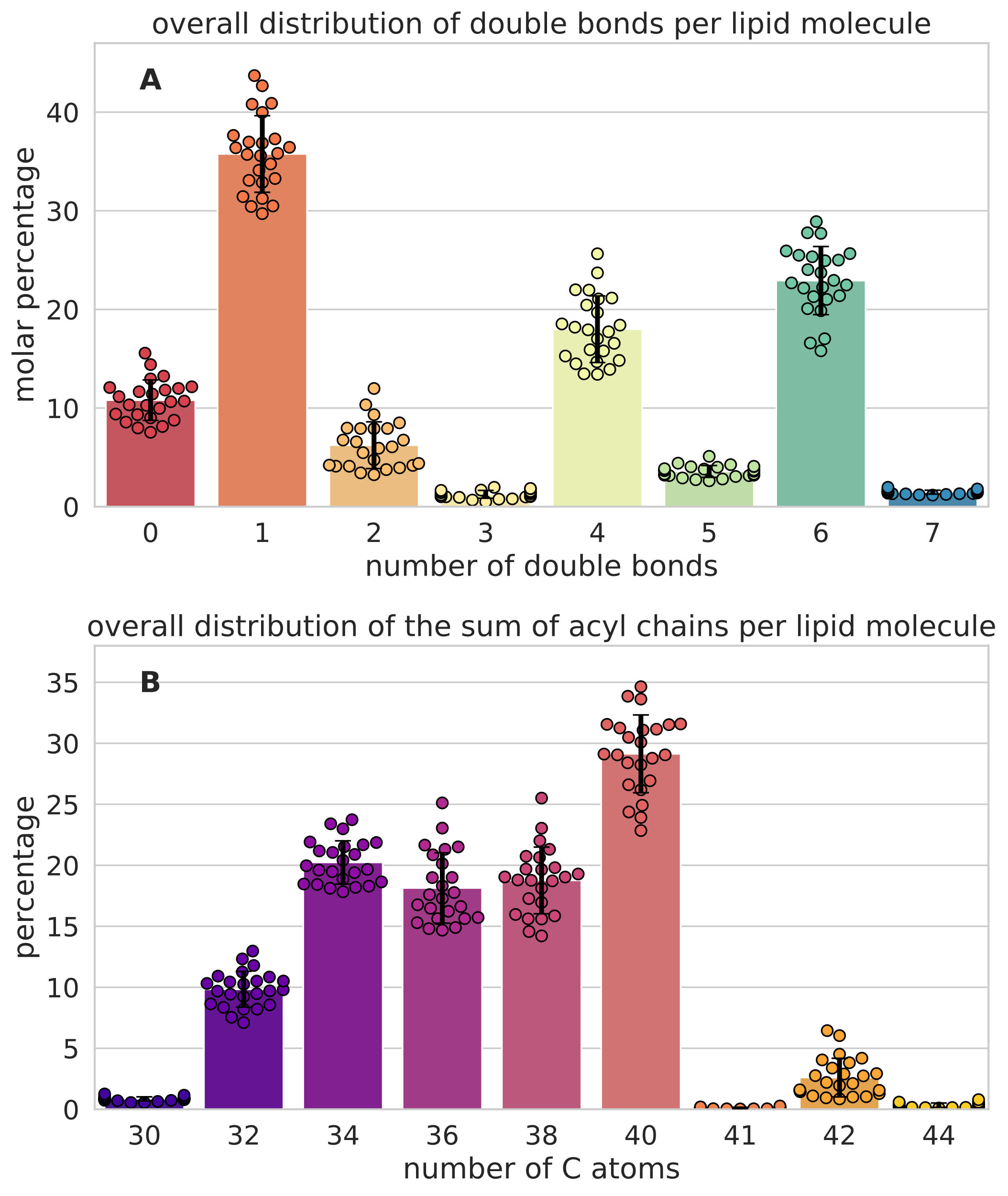


**Figure S3. A** The distribution of lipid unsaturation (number of C=C double bonds per lipid) across all samples (n=24) from all cases (n=16) as percentage of the total lipid content. **B** The distribution of the sum of acyl chains (number of C atoms) as percentage of the total lipid content.

**Distribution of the sum of acyl chains and double bonds among tissue groups**

**
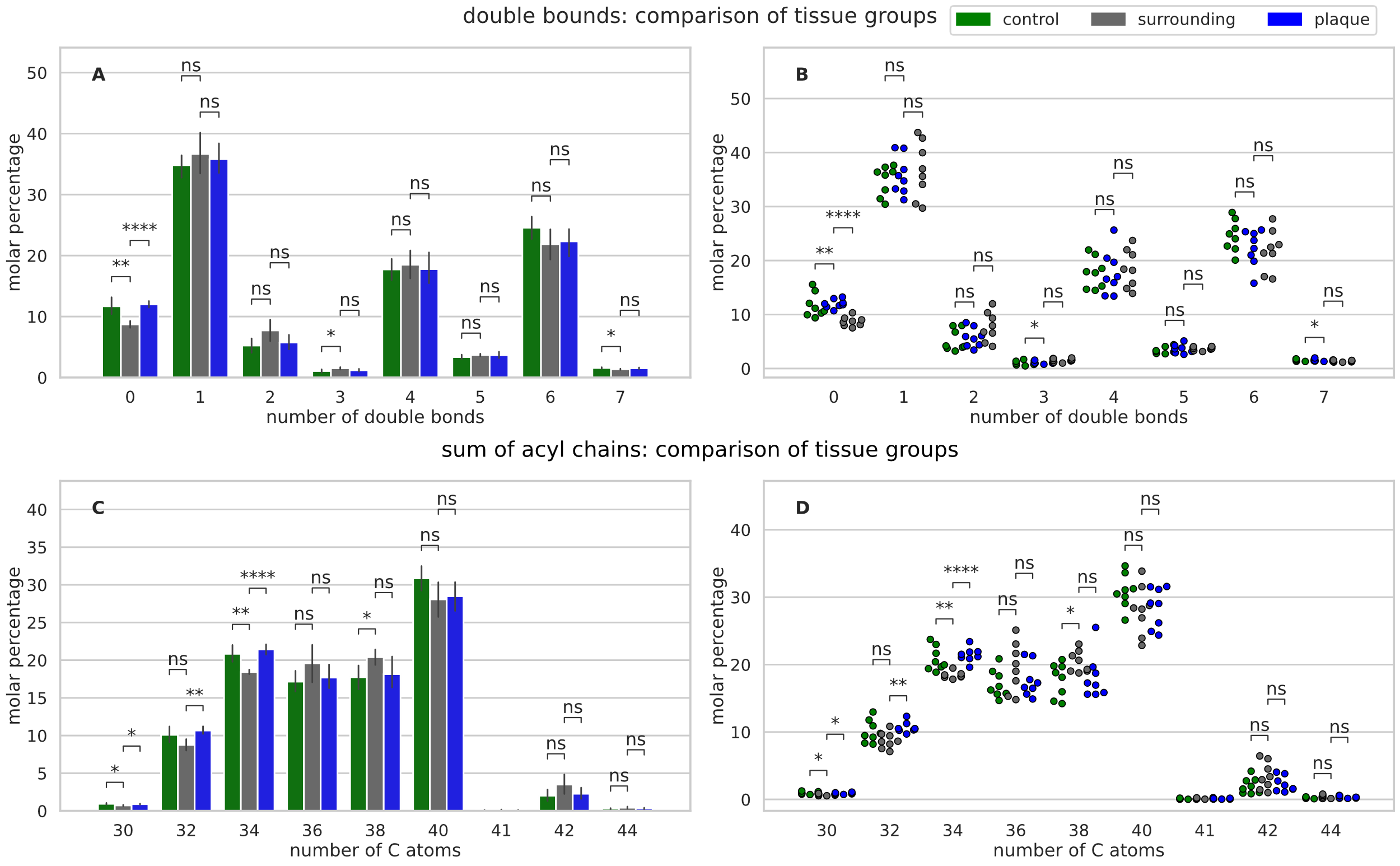
**

**Figure S4. Lipid unsaturation and sum of acyl chain composition** analyzed by flow injection analysis mass spectrometry (FIA-MS) of label-free extracted Aβ plaques, surrounding tissue, and gray matter from healthy controls (HC). Distribution of lipid unsaturation (number of C=C double bonds per lipid) across samples (n=24) from all cases (n=16) for the tissue groups (**A**) and individual samples (**B**), expressed as a percentage of total lipid content. And the distribution of FA length (number of C atoms in FAs per lipid molecule) across all samples (n=24) from all cases (n=16) and tissue groups (**C**) and individual samples (**D**) as percentage of the total lipid content. The comprehensive statistical reports for these figures correspond to those presented in Figures 3A and 4A and can be found in Table S2.

**Lipid class distribution**

**
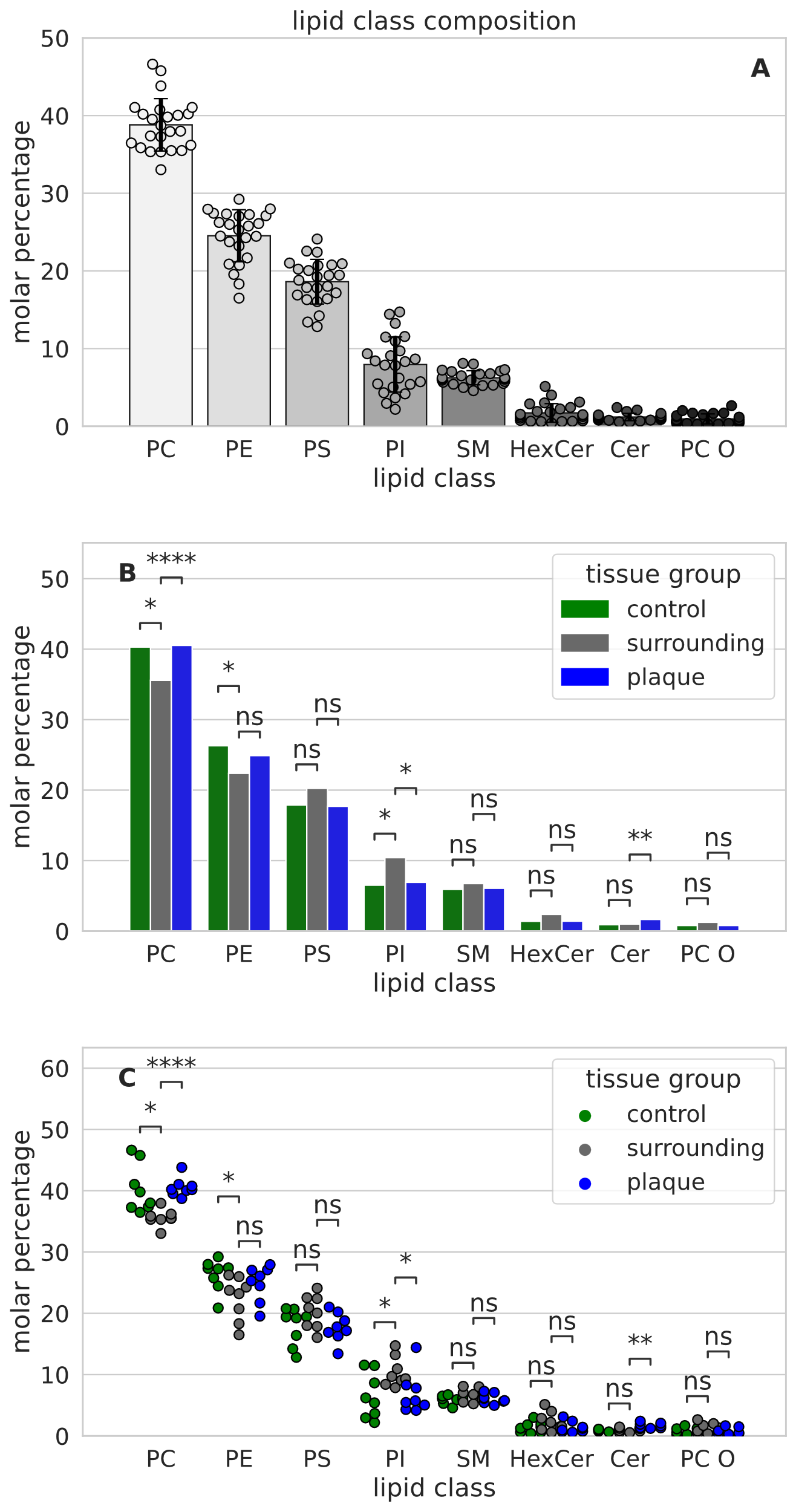
**

**Figure S5. Lipid class composition** derived by flow injection analysis mass spectrometry (FIA-MS) of label-free extracted Aβ plaques, surrounding tissue, and gray matter from healthy control (HC) cases. **A** The distribution of lipid classes across all samples (n=24) from all cases (n=16) as percentage of the total lipid content. **B** Some lipid classes differ significantly between the tissue groups. **C** Distribution of lipid classes across individual samples. The complete statistical reports, including p-values, t-values and degrees of freedom, can be found in Supplement S9.

**Phosphatidylcholine (PC) – separate analysis**

**
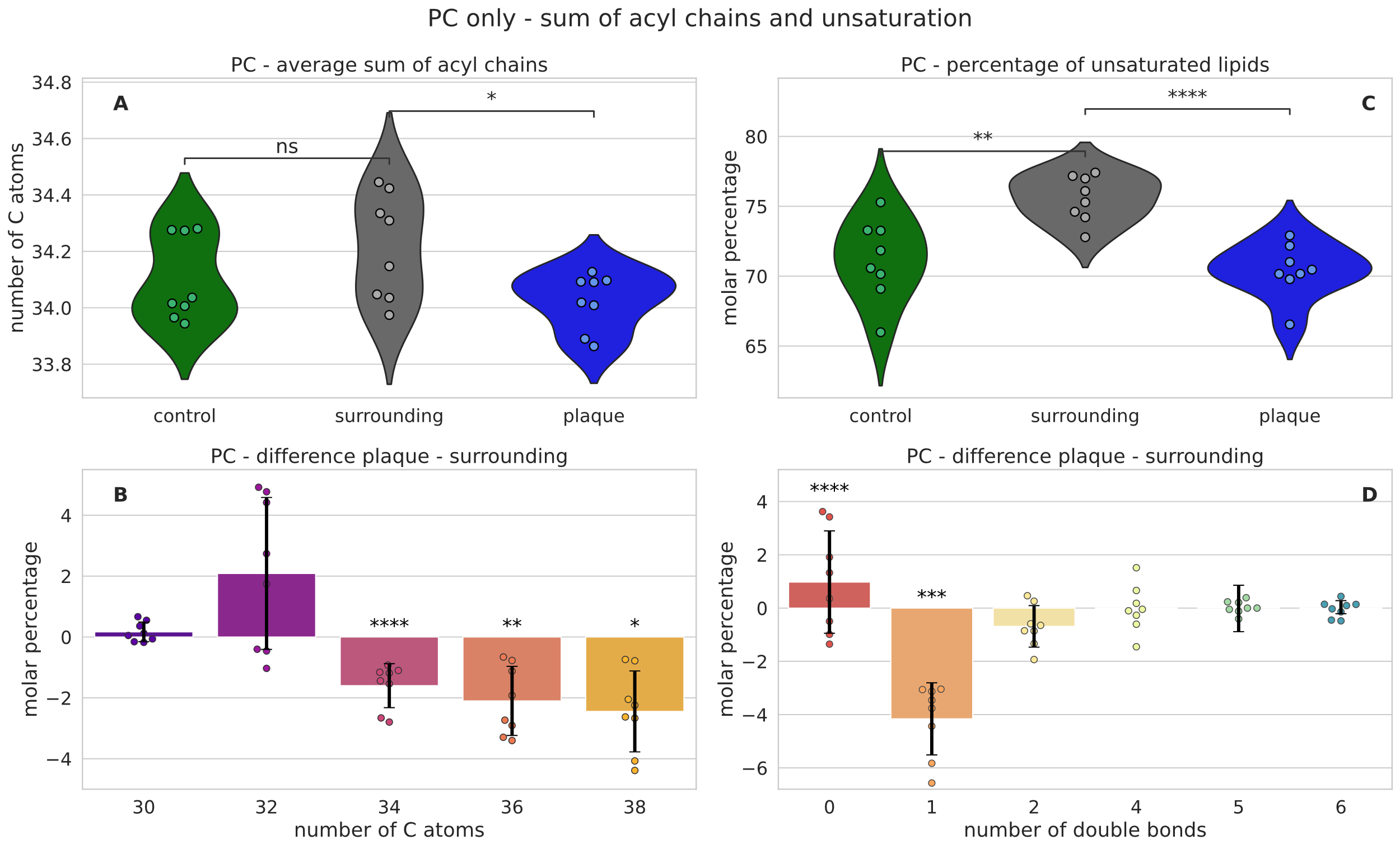
**

**Figure S6. Sum of acyl chains and lipid unsaturation of Phosphatidylcholine (PC) only** derived by flow injection analysis mass spectrometry (FIA-MS) of label-free extracted Aβ plaques, surrounding tissue, and gray matter from healthy control (HC) cases. **A** The average sum of acyl chains is significantly lower in plaques, compared to surrounding and control tissue. **B** The distribution difference between plaques and their surrounding tissue reveals that short FAs (<35 C atoms per lipid) are significantly increased in plaques, whereas long FAs (>35 C atoms per lipid) are significantly decreased. **C** The relative content of unsaturated lipids differs significantly between the tissue groups and is lowest in plaques. **D** The distribution difference of lipid unsaturation between plaques and their surrounding tissue reveals that unsaturated lipids are significantly increased in plaques, whereas unsaturated lipids are decreased. This effect is stronger than in the overall lipid composition. The full statistical reports, including p-values, t-values and degrees of freedom, can be found in Supplement S9.

**PCA Loadings**

**
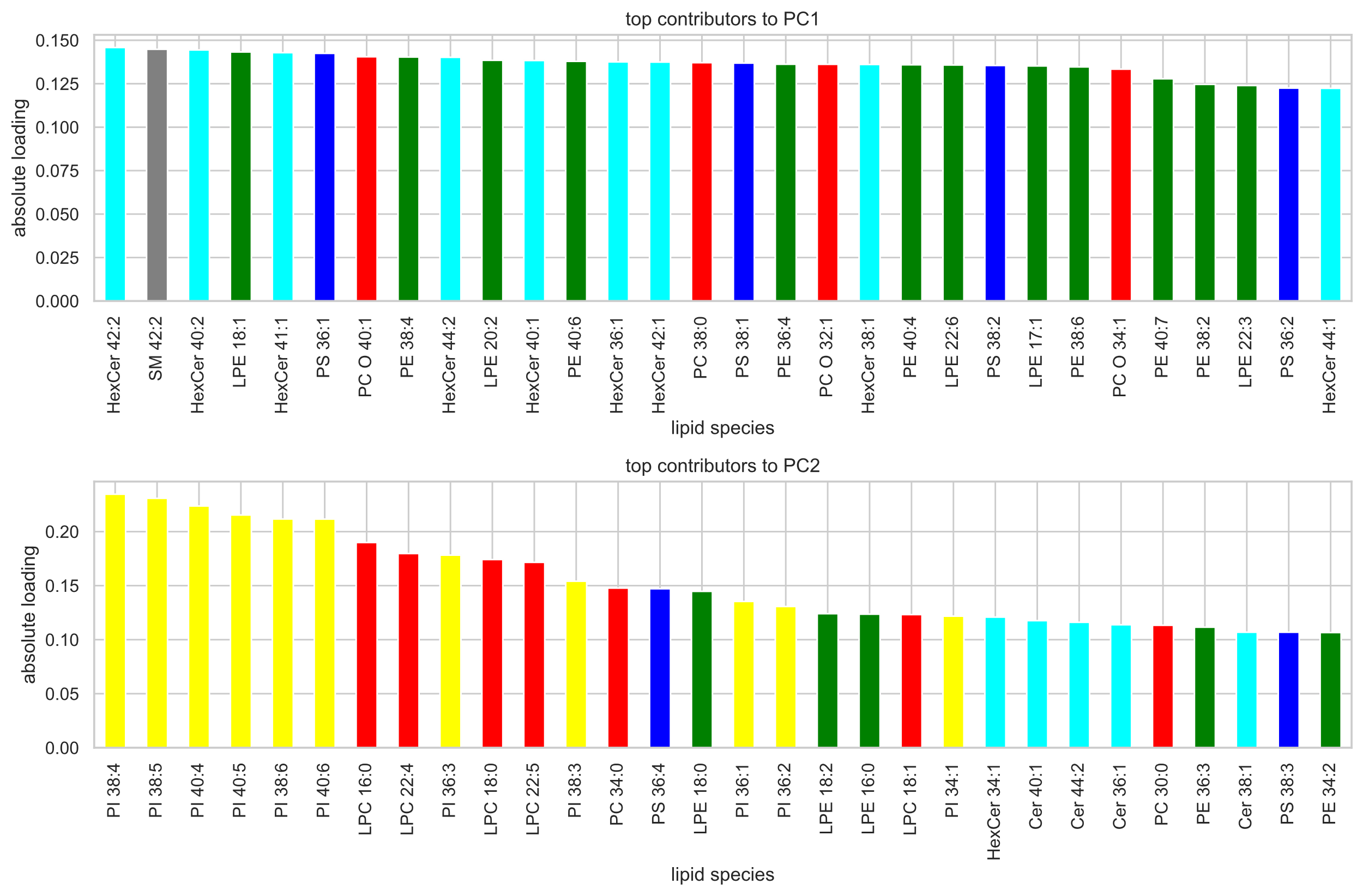
**

**Figure S7. Top 30 contributors of the first two PCA components.** PC1 is dominated by HexCer (cyan) and PE (green). PC2 is dominated by PI (yellow) and PC (red). For clarity, annotations have been simplified in the figure. However, note that Sphingolipids (Cer, HexCer, SM) should technically include “;O2” in their annotation.

**Fill lipid species correlation matrix**

**
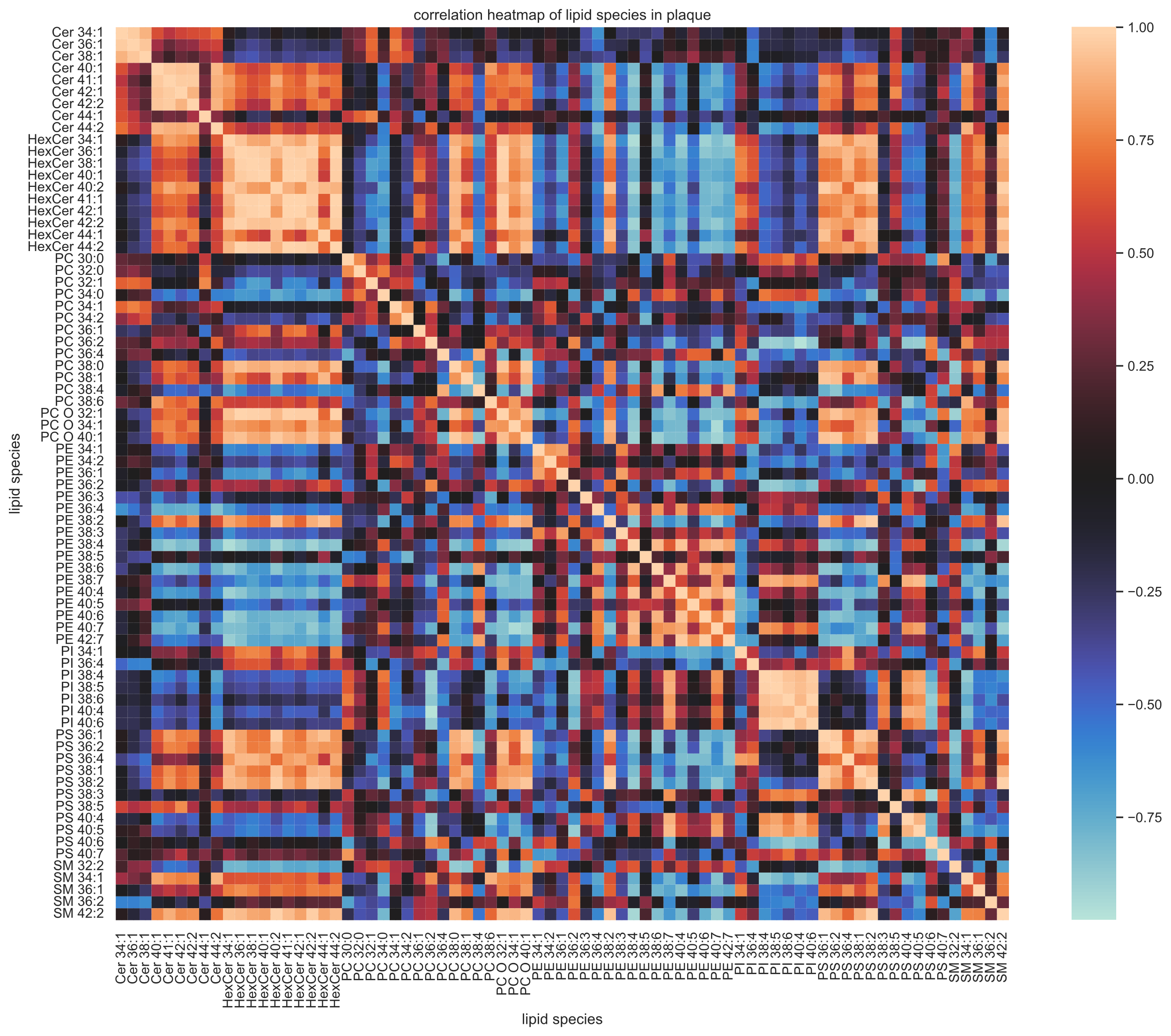
 Figure S8. Full correlation matrix** between all lipid species. Red indicates correlation, whereas blue indicates anti-correlation. For clarity, annotations have been simplified in the figure. However, note that Sphingolipids (Cer, HexCer, SM) should technically include “;O2” in their annotation.

**Full statistical reports from supplementary material**

| **Compared Groups** | **p-value** | **t-value** | **Degrees of freedom** |
| --- | --- | --- | --- |
| **Figure S5B / S5C: lipid class composition** | | |  |
| PC: surrounding vs. plaque | <.001 | -6.940 | 45.44 |
| PC: control vs. surrounding | .011 | 3.208 | 28.32 |
| PE: surrounding vs. plaque | .143 | -1.554 | 44:37 |
| PE: control vs. surrounding | .026 | 2.518 | 42.29 |
| PS: surrounding vs. plaque | .070 | 1.968 | 45.02 |
| PS: control vs. surrounding | .124 | -1.635 | 45.64 |
| PI: surrounding vs. plaque | .034 | 2.385 | 41.53 |
| PI: control vs. surrounding | .028 | -2.503 | 39.58 |
| SM: surrounding vs. plaque | .181 | 1.415 | 42.50 |
| SM: control vs. surrounding | .090 | -1.846 | 39.10 |
| HexCer: surrounding vs. plaque | .158 | 1.513 | 37.01 |
| HexCer: control vs. surrounding | .149 | -1.555 | 35.83 |
| Cer: surrounding vs. plaque | .005 | -3.344 | 43.64 |
| Cer: control vs. surrounding | .504 | -0.694 | 32.04 |
| PC O: surrounding vs. plaque | .218 | 1.302 | 39.03 |
| PC O: control vs. surrounding | .213 | -1.315 | 38.57 |
| **Figure S6A: PC - average sum of acyl chains** | | |  |
| surrounding vs. plaque | .027 | 2.563 | 10.69 |
| control vs. surrounding | .195 | -1.364 | 13.37 |
| **Figure S6B: PC – difference plaque - surrounding** | | |  |
| 34 | <.001 | -7.723 | 13.44 |
| 36 | .002 | 3.765 | 12.80 |
| 38 | .020 | 2.740 | 10.78 |
| **Figure S6C: PC – percentage of unsaturated lipids** | | |  |
| surrounding vs. plaque | <.001 | 5.826 | 13.71 |
| control vs. surrounding | .003 | -3.727 | 11.07 |
| **Figure S6D: PC – difference plaque - surrounding** | | |  |
| 0 | <.001 | -5.826 | 13.71 |
| 1 | <.001 | 5.555 | 10.96 |

**Table S9.** **Full statistical reports for supplementary material:** All analyses utilized the Welch t-test for the computation of p-values, t-values and degrees of freedom across all datasets.
